# Nutrition, Inflammation, and Acute Pancreatitis

**DOI:** 10.1155/2013/341410

**Published:** 2013-12-29

**Authors:** Max Petrov

**Affiliations:** Department of Surgery, The University of Auckland, Private Bag 92019, Auckland 1142, New Zealand

## Abstract

Acute pancreatitis is acute inflammatory disease of the pancreas. Nutrition has a number of anti-inflammatory effects that could affect outcomes of patients with pancreatitis. Further, it is the most promising nonspecific treatment modality in acute pancreatitis to date. This paper summarizes the best available evidence regarding the use of nutrition with a view of optimising clinical management of patients with acute pancreatitis.

## 1. Epidemiology and Economic Burden of Acute Pancreatitis

Acute pancreatitis is a common digestive disease and the most frequent disorder of the pancreas. It is observed in every part of the world, but the incidence of acute pancreatitis varies considerably between countries. Relatively low figures are reported from the United Kingdom (9.8 cases per 100,000 population per year), Germany (13.1 cases per 100,000 population per year), and Japan (15.4 cases per 100,000 population per year) [1–3]. Medium figures are reported from New Zealand (29.3 cases per 100,000 population per year), Iceland (32.3 cases per 100,000 population per year), and Norway (34.4 cases per 100,000 population per year) [4–6]. The highest figures in the literature are reported from the United States (73.0 cases per 100,000 population per year) and Finland (73.4 cases per 100,000 population per year) [7, 8]. However, direct comparison of incidences between countries is hardly legitimate because of diagnostic, aetiological, ethnic, and other differences between the study populations.

Several reports from the United States and Western Europe indicate that the frequency of this disease has increased dramatically [9–11]. In the United States, there were significant upward trends in both absolute numbers of hospitalisations for acute pancreatitis and annual incidence [7]. The absolute number of admissions was 101,000 in 1988 as compared with 201,000 in 2002. The annual incidence was also the lowest in 1988 at 41 cases per 100,000 population and peaked in 2002 at 73 cases per 100,000 population. In Denmark, the annual incidence increased from 17 cases per 100,000 population in 1981 to 32 cases per 100,000 population in 2000 [9]. Similarly, in Sweden, the annual incidence increased from 18 cases per 100,000 population in 1985 to 35 cases per 100,000 population in 1999 [10].

Most studies reporting on trends also indicate a steady decrease in the case-fatality rate over time. The case fatality for acute pancreatitis has decreased from 15%–21% in the earlier studies to 2%–7% in the recent studies [1, 12]. Although the case-fatality rate has decreased, several studies have reported that the population mortality rate has remained unchanged over time. The likely explanation for this is that, given that the case-fatality rate is a proportion of deaths within a designated population of people with acute pancreatitis and the population mortality is a rate per 100,000 population, better detection of mild cases within a population results in a decrease in case fatality but not in the population mortality rate. In addition, a proportional increase in the number of nonmild acute pancreatitis cases from an increase in the incidence may be offset by a decrease in mortality from earlier recognition and better treatment of local and systemic complications over time [13–15].

Patients with acute pancreatitis also pose a considerable financial burden to health care systems. An earlier study of patients with necrotising pancreatitis from the United Kingdom estimated that the actual cost of treatment ranged from *£*9296 to *£*33796, of which two-thirds was attributable to hospitalisation, 20% to surgical and endoscopic interventions, and 16% to investigations [1]. A recent study from the United States estimated that the total cost of acute pancreatitis admissions in 2003 was $2.2 billion (95% confidence interval 2.0–2.3 billion). Further, mean cost per hospitalisation was $9870 (95% confidence interval 9300–10,400) and mean cost per hospital day was $1670 (95% confidence interval 1620–1720) [9].

## 2. Role of Enteral Nutrition in Curtailing Inflammation of the Pancreas

Acute pancreatitis is a common disease with an increasing incidence. Still high morbidity and mortality in this disease as well as the overwhelming cost of treatment indicate significant room for improvement in clinical management. While there is no specific therapy for patients with acute pancreatitis as yet, adequate early treatment with established non-specific modalities has led to improved outcomes [16–19]. There have been several recent advances in the early non-specific management of acute pancreatitis. These include emergence of randomised controlled trials on fluid resuscitation and analgesia, more data (albeit conflicting) on the prophylactic use of antibiotics, and restriction of indications for early therapeutic endoscopic retrograde cholangiopancreatography to patients with coexisting acute cholangitis. However, the most notable and consistent improvement in outcomes over the last decade has come from the use of enteral nutrition in patients with acute pancreatitis [20–22].

The importance of providing nutritional support in patients with acute pancreatitis has been known since the 1970s. Parenteral nutrition was regarded as the standard of nutritional management for nearly four decades due to the advocacy of the “pancreatic rest” concept. The rationale for this concept was to rest the inflamed pancreas, thereby preventing stimulation of exocrine function and release of proteolytic enzymes. However, critics argued that, in addition to cost and catheter-related sepsis, parenteral nutrition might lead to electrolyte and metabolic disturbances, gut barrier alteration, and increased intestinal permeability. Comparison of total parenteral nutrition and total enteral nutrition in patients with predicted severe acute pancreatitis was the subject of eight randomised controlled trials (Table 1) [23–30]. The results were statistically aggregated in several meta-analyses, all of which demonstrated the benefits of enteral over parenteral nutrition. In particular, a meta-analysis of high-quality randomised controlled trials only has shown a significant 2.0-fold reduction in the risk of total and pancreatic infectious complications and a 2.5-fold reduction in the risk of death in patients receiving total enteral nutrition [31–33].

Despite the evident clinical benefits of enteral over parenteral nutrition in terms of the reduction in risk of infectious complications and mortality, the exact mechanism of its favourable effect remains unclear [34–36]. It is believed that enteral nutrition may prevent or attenuate the mucosal barrier breakdown and subsequent bacterial translocation that play a pivotal role in the development of infectious complications in the course of severe acute pancreatitis. When monitoring mucosal barrier function, permeability of the structural mucosal barrier is often the main parameter measured. Unfortunately, there is no consistency in the clinical studies with regard to gut permeability. On the one hand, three clinical studies of acute pancreatitis showed increased intestinal permeability to both micromolecules and macromolecules in patients with predicted severe acute pancreatitis when compared with predicted mild acute pancreatitis and healthy volunteers [37–39]. On the other hand, the randomised controlled trial by Powell and colleagues, in which patients with predicted severe acute pancreatitis were randomised to receive either enteral nutrition or no artificial nutritional support, showed significantly increased intestinal permeability by day 4 in patients allocated to the enteral nutrition group [40]. Similarly, the randomised controlled trial of nasogastric versus parenteral feeding in predicted severe patients by Eckerwall and colleagues demonstrated impaired gut permeability on day 3 in the enteral nutrition group [41]. However, in fact, both randomised controlled trials included a considerable number of patients with mild acute pancreatitis (11 of 27 and 26 of 48, resp.), in which it is unlikely that intestinal permeability changed considerably.

Furthermore, concentrations of anti-endotoxin core antibodies for immunoglobulin M were also used as an indirect marker for intestinal permeability. Results of the randomised controlled trial by Windsor and colleagues showed that serum immunoglobulin M antibodies decreased significantly following 7 days of enteral nutrition when compared with the parenteral nutrition group (*P* < 0.05) [42]. Similarly, the randomised controlled trial by Gupta and colleagues demonstrated that immunoglobulin M antibodies fell significantly in the enteral nutrition group (*P* = 0.03) and tended to rise in the parenteral nutrition group over the week of treatment [43]. Conversely, the randomised controlled trial by Eckerwall and colleagues found decreasing levels of immunoglobulin M antibodies in both the enteral nutrition and parenteral nutrition groups, with no significant difference at any time point during ten days of observation [41]. The mechanism of beneficial influence of enteral nutrition in acute pancreatitis warrants further investigation, and more studies on the use of enteral nutrition in patients with acute pancreatitis are needed.

## 3. Optimal Route of Enteral Nutrition Delivery

The previous section has demonstrated that enteral nutrition is preferred to parenteral nutrition because it leads to significantly better glycemic control, decreases infectious complications, reduces the need for surgery, and reduces mortality. With these benefits apparent, one of the unanswered questions has been to determine if there is an optimal site for tube placement during feeding administration. The alternatives include postpyloric (mainly, nasojejunal) and prepyloric (nasogastric) tube placement. The former usually requires the assistance of an endoscopist or a radiologist, and this may result in a delay in commencing enteral nutrition. This delay may have an impact on the clinical outcome because it is now believed that enteral nutrition should commence as soon as possible after adequate fluid resuscitation in order to maximise clinical benefit. In contrast, a nasogastric feeding tube can usually be inserted immediately and with ease, such that prepyloric feeding can be started without delay.

The question of optimal site of enteral feeding in acute pancreatitis also relates to the “pancreatic rest” concept. The central tenet of this concept is that enteral nutrition delivered into any part of the upper gastrointestinal tract other than the jejunum stimulates pancreatic secretion and, consequently exacerbates the severity of acute pancreatitis [44–46]. Given that this concept remained unchallenged for decades, the majority of clinical studies in the field of acute pancreatitis were conducted using nasojejunal tube feeding. However, accumulating evidence from other fields, particularly critical care medicine, suggests that nasogastric feeding may be as safe and effective as nasojejunal feeding, at least in some patients. Thus, there is a need and justification for exploring questions concerning the optimal route of enteral nutrition delivery to be used in patients with acute pancreatitis.

A number of randomised controlled trials and the latest meta-analysis have demonstrated the equivalence of nasogastric and nasojejunal tube feeding in terms of safety and tolerance in critically ill patients [47, 48]. While this may be true for this group of patients, it is recognised that patients with acute pancreatitis are particularly prone to gastric ileus because of the subjacent inflamed pancreas. This has been given as a reason for preferentially providing enteral nutrition into the jejunum [49–51]. Another reason given is to avoid the provision of enteral nutrition proximal to the jejunum where there is concern that it might induce exocrine pancreatic stimulation and consequently a risk of increased severity of acute pancreatitis. Most studies in patients with acute pancreatitis have employed nasojejunal tube feeding, but there are some studies that employed nasogastric tube feeding.

Two systematic literature reviews on nasogastric feeding in acute pancreatitis are available in the literature. The first review attempted to define the feasibility of this route of nutrition by meta-analyzing the data from randomised controlled trials of nasogastric versus “conventional” nutrition [52]. The pooled estimates and variance of the treatment effect were based on the statistical aggregation of the results from studies with essentially different comparators, that is, total parenteral feeding and nasojejunal tube feeding. Such an approach might be misleading because parenteral feeding is no longer considered the first-line approach in acute pancreatitis. Moreover, there was a marked heterogeneity in baseline risk among the studies included in that meta-analysis, particularly in regard to age and gender ratio, and incorrect pooled estimates were presented due to inaccurate data input. Furthermore, that review did not determine the safety, tolerance, and efficacy of nasogastric tube feeding alone.

The second review aimed to determine the safety and tolerance of nasogastric tube feeding alone and to assess the relative efficacy of nasogastric versus nasojejunal feeding in patients with acute pancreatitis [53]. This was done by analyzing all of the literature (randomised and nonrandomised studies) relating to acute pancreatitis and use of nasogastric tube feeding. A computerised literature search of the Cochrane Central Register of Controlled Trials, EMBASE, and MEDLINE was conducted. The search strategy for the Cochrane Central Register of Controlled Trials was “acute pancreatitis” and “nutrition.” The search strategy for EMBASE included the terms “acute pancreatitis” and “enteral nutrition” or “enteral feeding.” The search strategy for MEDLINE was “acute pancreatitis” (title/abstract) and “enteral nutrition” (title/abstract) or “enteral feeding” (title/abstract). No language restrictions were applied. From the studies on enteral nutrition in acute pancreatitis, only data on patients receiving enteral feeding formula via nasogastric tube were extracted. Bibliographies of all selected articles that included information on nasogastric tube feeding in acute pancreatitis were reviewed for other relevant articles. The following selection criteria were used to identify published studies for inclusion in this systematic review:

- study design—cohort study or randomised controlled trial;
- population—patients with acute pancreatitis;
- intervention—nasogastric tube feeding;
- outcome—at least one of the following outcomes: tolerance, organ failure, infectious complications, and mortality.

A total of 397 publications were identified using the above search strategy. Of these, 392 articles did not meet the inclusion criteria and were subsequently excluded. A total of five studies were included in this systematic review. One study was a cohort study, whereas four other studies were randomised controlled trials.  Table 2 demonstrates the characteristics of studies included in this review [54–57]. All the studies were conducted in patients with predicted severe acute pancreatitis (as defined by the authors). Overall, 131 patients who received nasogastric tube feeding were identified from these studies. The severity of the patients at admission was comparable in all five cohorts, based on Acute Physiology And Chronic Health Evaluation II scoring.  Table 3 presents the baseline characteristics of patients who received nasogastric tube feeding.

Nasogastric feeding-related outcomes, including safety and tolerance, are presented in Table 4. Full tolerance was achieved in 107 of 131 (82%) patients who did not require temporary reduction, stoppage, or withdrawal of nasogastric feeding. The 24 patients who had a modification of the nasogastric tube feeding regimen presented signs of gastric ileus (*n* = 7) and troublesome diarrhoea (*n* = 14) or repeatedly removed their feeding tube (*n* = 3).

The other clinically meaningful outcomes of the studies are summarised in Table 5. Sixty-one of 92 (45%) patients required ventilatory support. There was no evidence of aspiration pneumonia in any of the patients. Infected pancreatic necrosis was revealed in 15 (12%) patients. Multiple organ failure developed in 21 (16%) patients. The mortality rate was 15%.

The meta-analysis was restricted to randomised studies of nasogastric versus nasojejunal feeding [55–57]. In three eligible trials, a total of 82 patients received enteral nutrition via the nasogastric route and 75 patients via the nasojejunal route. The use of nasogastric feeding resulted in a nonsignificant reduction in the risk of death (relative risk 0.71; 95% confidence interval 0.38 to 1.32; *P* = 0.28). The number of nutrition-associated adverse events was similar between the two groups. As a consequence, nasogastric feeding was associated with a nonsignificant increase in the risk of troublesome diarrhoea (relative risk 1.39; 95% confidence interval 0.57–3.36; *P* = 0.47) and a nonsignificant decrease in the risk of pain relapse following feeding (relative risk 0.84; 95% confidence interval 0.27–2.59; *P* = 0.76). Overall, patients in both groups did not differ significantly in terms of intolerance to feeding (relative risk 1.23; 95% confidence interval 0.59–2.55; *P* = 0.57). There was no heterogeneity between the study results for all comparisons (*I* ^2^ = 0%).

This systematic review has demonstrated the safety and tolerance of nasogastric tube feeding in at least four out of five patients with acute pancreatitis. The study population was limited to patients with a predicted severe course of acute pancreatitis and the clinical outcomes were within the expected range for this category of patients. Nasogastric tube feeding-related problems occurred in less than 20% of patients and they were relatively minor. There were no recorded cases of aspiration pneumonia.

Three randomised controlled trials included in the meta-analysis consistently yielded no tangible difference between nasogastric and nasojejunal feeding in terms of safety and tolerance [55–57]. It should be acknowledged that the trials had some flaws. In particular, it was argued that it is likely that jejunal feeding in the trial from Glasgow was actually duodenal (because true jejunal placement would have been difficult with the types of feeding tubes and placement techniques used), meaning that both feeding arms may have caused equivalent stimulation of pancreatic secretion [58]. The shortcoming of the randomised controlled trial by Kumar and colleagues was that there was a considerable delay (7.8 ± 6.5 and 5.7 ± 4.7 days after symptom onset in the nasogastric and nasojejunal groups, resp.) and that enteral nutrition was commenced late [59]. In addition, the authors observed a high mortality (31% and 29% in the nasogastric and nasojejunal groups, resp.) which might reflect the tendency towards conservative management of patients with infected pancreatic necrosis. The randomised controlled trial by Singh and colleagues suffered from the same shortcoming; that is, the feeding protocol in both groups was commenced relatively late (10 [4–21, 60, 61] and 11 [3–30, 34–39, 44–51, 60–63] days after symptom onset in the nasogastric and nasojejunal groups, resp.) [64]. Apart from these concerns, the three randomised controlled trials were insufficiently powered individually to detect any difference or to demonstrate equivalence between the studied groups in terms of mortality. An adequately powered randomised controlled trial would need to enrol nearly 200 patients per arm in order to show a decrease in mortality from 14% (average rate in the nasogastric group in the present review) to 6% (best results in the nasojejunal group of randomised controlled trials on enteral versus parenteral nutrition) with 80% power and *α* = 0.05 (two-sided). Such a sample size is appreciably large, even for a multicentre study.

Another relevant issue in considering nasogastric tube feeding is the effect on exocrine pancreatic function. It was shown by O'Keefe and colleagues that all forms of enteral nutrition stimulate pancreatic secretion [65, 66]. In particular, when compared with placebo saline, an oral liquid polymeric diet resulted in a significantly higher level of amylase (*P* < 0.01) and lipase (*P* < 0.01); a duodenal polymeric enteral formula led to increased levels of amylase (*P* < 0.01), lipase (*P* < 0.01), and trypsin (*P* < 0.01); and a duodenal elemental feeding formula resulted in an elevated level of lipase (*P* < 0.05). The same research group also compared the pancreatic secretory response to tube feeding delivered into the duodenum and the mid (40–60 cm distal to the ligament of Treitz) and distal (100–120 cm distal to the ligament of Treitz) jejunum [67, 68]. Even though the authors did not find a direct relationship between the decrease in enzyme secretion and distance down the mid-distal jejunum, they demonstrated significantly lower secretion of trypsin (*P* < 0.01) and lipase (*P* < 0.05) in response to the elemental formula delivered into the jejunum (40 cm or more distal to the ligament of Treitz) in comparison with the duodenum. Moreover, the trypsin and lipase secretory response in the mid-distal jejunum group was as low as in the control group (fasting).

However, it should be noted that these studies of the effects of enteral feeding on exocrine pancreatic function were in healthy subjects. There is now convincing evidence that patients with acute pancreatitis have significantly lower rates of enzyme secretion compared with healthy subjects [68]. Furthermore, when patients with mild-to-moderate acute pancreatitis were compared with those with severe acute pancreatitis, a lower secretion of trypsin (6-fold), amylase (22-fold), and lipase (42-fold) was found in the latter group, suggesting that pancreatic enzyme secretion is inversely related to the severity of acute pancreatitis. In line with this finding, another study showed an 86% rate of pancreatic exocrine insufficiency (measured by faecal pancreatic elastase-1) in patients recovering from severe attacks of acute pancreatitis [69]. Moreover, the severity of pancreatic exocrine insufficiency correlated with the extent of pancreatic necrosis. These data suggest that injured acinar cells are not able to respond fully to the physiological stimuli of secretion which may go some way towards explaining the findings of this study that, contrary to popular belief, nasogastric tube feeding does not appear to aggravate the severity of acute pancreatitis [53].

## 4. Optimal Enteral Nutrition Formulation

The previous section has challenged the notion of putting the pancreas at rest by showing that tube feeding into the stomach is safe and well tolerated in the vast majority of patients with acute pancreatitis. However, it has been known since the groundbreaking experiments by Ivan Pavlov and his disciples that not only the site of feeding but also the composition of enteral feed may affect the pancreatic secretory response and, thus, the question of the optimal enteral nutrition formulation is important, both in the management of patients with acute pancreatitis and for the validity of the “pancreatic rest” concept.

The “pancreatic rest” concept has been regarded as a key element in the early management of patients with acute pancreatitis. As a consequence, for decades, these patients have received total parenteral nutrition in an attempt to avoid stimulation of pancreatic enzyme secretion. However, over the last decade, a number of randomised controlled trials have consistently shown the superiority of enteral over parenteral nutrition in terms of reducing the rate of infectious complications and death [20, 25–27]. Further, a recent meta-analysis of randomised controlled trials established the absolute value of enteral nutrition by demonstrating significantly reduced mortality in patients with acute pancreatitis who received enteral nutrition in comparison with those who did not receive any kind of nutrition [88].

Now that the benefits of enteral nutrition in patients with acute pancreatitis have become widely accepted, one of the key questions to answer is what is the optimal formulation to use [89]. There are more than 100 different enteral nutrition formulations available. These can be broadly classified into the following categories:

- elemental—comprising amino acids or oligopeptides, maltodextrins, and medium—chain and long-chain triglycerides;
- polymeric—comprising nonhydrolyzed proteins, maltodextrins, and oligofructosaccharides, as well as long-chain triglycerides;
- immune-enhancing—comprising substrates that have been hypothesised to modulate the activity of the immune system, for example, immunonutrition (glutamine, arginine, and omega-3 fatty acids), probiotics, fibre-enriched formulation.

In patients with acute pancreatitis, the use of elemental over polymeric formulations presents a number of theoretical advantages because it is believed that an elemental formulation has superior absorption from the intestine, stimulates pancreatic secretions to a lesser degree, and is better tolerated [35, 90, 91]. On the other hand, the major disadvantage of an elemental formulation is its cost, which is reportedly 3–7-fold higher than that of a polymeric formulation. The cost of an immune-enhancing formulation is 3–5-fold higher than the cost of an elemental formulation, but whether this leads to better clinical outcomes is unknown [34, 47, 92–94]. Both elemental and immune-enhancing formulations have a higher osmolar load than polymeric formulations, which are isomolar and so may cause diarrhoea. In addition, increased mortality associated with the use of probiotics in patients with acute pancreatitis, observed in the recently published PROPATRIA trial, has highlighted the need for careful selection of enteral nutrition formulations in current clinical practice as well as in future basic and clinical research [31, 95].

A recent comprehensive systematic literature review has compared the safety, tolerance, and efficacy of all enteral nutrition formulations used in randomised controlled trials of patients with acute pancreatitis [89]. Potentially relevant studies were identified using electronic and manual searches. An electronic search was performed in Scopus, Cochrane Controlled Clinical Trials Register, and MEDLINE (searched through PubMed) databases using the terms “acute pancreatitis,” “enteral nutrition,” “glutamine,” “arginine,” “omega-3 fatty acids,” “probiotics,” and “dietary fibre.” Results were limited to trials in humans. This was also supplemented by scanning the bibliographies of retrieved articles and conference proceedings of selected scientific meetings (Digestive Disease Week, United European Gastroenterology Week, International Pancreatic Association, American Pancreatic Association, and European Pancreatic Club). All languages and types of publications were considered eligible.

In order to be included in the systematic review, a study had to

- be a randomised controlled trial in patients with acute pancreatitis;
- compare two different feeding regimens, at least one of which had to include enteral tube feeding (with type of the nutritional formulation used clearly specified);
- report on feeding intolerance (defined as an episode of temporary reduction, stoppage, or withdrawal of feeding) and at least one of the following outcomes: total infectious complications and in-hospital mortality.

Studies investigating the tolerance of oral refeeding or combined enteral and parenteral nutrition or postoperative nutrition were excluded.

The titles and abstracts of 384 identified papers were screened and 348 were excluded after initial screening. Sixteen publications were subsequently excluded: four were on refeeding in patients with acute pancreatitis, four were republished in a non-English language, three were conducted in patients with acute pancreatitis after surgery, two compared nasogastric and nasojejunal routes of enteral nutrition, two studied enteral nutrition supplemented with parenteral nutrition, and one used the same study population as in another included randomised controlled trial. Thus, a total of 20 randomised controlled trials met all the inclusion criteria [32, 33, 36, 41, 43, 46, 62, 70–73, 75, 93–104]. All included articles were published in peer-reviewed journals.

Among the 20 included randomised controlled trials, 19 were single-centre trials and there was one multicentre trial. Patients received an elemental formulation in eight arms of the included trials, a polymeric formulation in seven arms, a fibre-enriched enteral formulation in six arms, enteral nutrition supplemented with probiotics in four arms, and immunonutrition (glutamine, arginine, and omega-3 fatty acids) in three arms.

The 20 randomised controlled trials comprised a total of 1070 patients with acute pancreatitis (825 with predicted severe and 245 with predicted mild course of acute pancreatitis) [32, 33, 36, 41, 43, 46, 62, 70–73, 75, 93–104]. Twelve studies were limited to patients with predicted severe acute pancreatitis only.  Table 6 details the study characteristics of included trials.

### 4.1. Elemental versus Polymeric Formulation

One randomised controlled trial directly compared an elemental formulation with a polymeric formulation in 30 patients with mild or severe acute pancreatitis [104]. Given that direct meta-analysis was not possible, the two formulations were compared using the methodology of indirect adjusted meta-analysis. A total of 10 randomised controlled trials comprising 428 patients compared elemental and polymeric formulations indirectly, using parenteral nutrition as a reference treatment. In all patients with acute pancreatitis, the use of an elemental formulation did not result in a significant difference in risk of infectious complications (indirectly estimated relative risk 0.48; 95% confidence interval 0.06–3.76; *P* = 0.482) and death (indirectly estimated relative risk 0.63; 95% confidence interval 0.04–9.86; *P* = 0.741). The risk of feeding intolerance did not differ significantly between the two formulations (indirectly estimated relative risk 0.62; 95% confidence interval 0.10–3.97; *P* = 0.611). These effects were nonsignificant when only patients with severe acute pancreatitis were considered (Table 7).

### 4.2. Fibre-Enriched Formulation Supplemented with Probiotics versus Fibre-Enriched Formulation

A total of three randomised controlled trials comprising 403 patients directly compared a fibre-enriched formulation supplemented with probiotics and a fibre-enriched formulation only [70, 93, 95]. In all patients with acute pancreatitis, the use of probiotics did not result in a significant difference in the risk of infectious complications (relative risk 0.71; 95% confidence interval 0.40–1.27; *P* = 0.250) or death (relative risk 0.85; 95% confidence interval 0.18–4.14; *P* = 0.850). The risk of feeding intolerance did not differ significantly between the two formulations (relative risk 0.69; 95% confidence interval 0.43–1.09; *P* = 0.110). These effects were nonsignificant when only patients with severe AP were considered (Table 7).

### 4.3. Additional Studies Not Included in the Meta-Analyses

Four randomised controlled trials were not included in the above meta-analyses because they were not able to be compared, directly or indirectly, with any other randomised controlled trials. One randomised controlled trial compared an elemental formulation supplemented with probiotics versus parenteral nutrition [94]. The use of an enteral feeding formulation resulted in a significantly reduced rate of septic complications (eight of 36 (22%) versus 21 of 38 (55%) patients; *P* = 0.008) and no difference in mortality (no deaths in both groups). Another trial compared a fibre-enriched formulation with a fibre-free formulation and demonstrated no difference in infectious complications (two of 15 (13%) patients in each group) or mortality (two of 15 (13%) patients versus four of 15 (27%) patients) [85]. All patients tolerated the fibre-enriched formulation, whereas feeding intolerance was observed in two patients who received the fibre-free formulation. One trial compared a polymeric formulation with no nutrition [62]. There was no difference between the groups with regard to rate of infectious complications or mortality. A final randomised controlled trial compared a polymeric formulation supplemented with n-3 polyunsaturated fatty acids with a polymeric formulation only [71]. There was no significant difference between the groups with regard to rate of infectious complications (five of 14 (36%) versus seven of 14 (50%) patients) or mortality (one of 14 (7%) versus two of 14 (14%) patients).

The major finding of this systematic literature review was that the use of a polymeric, in comparison with an elemental, enteral nutrition formulation was not associated with a statistically significant difference in tolerance of feeding, or risk of infectious complications and mortality [89]. In addition, it shows that a fibre-enriched formulation may be safely administered in patients with acute pancreatitis and its supplementation with immunonutrition or probiotics does not improve clinically meaningful outcomes.

This systematic literature review further questioned the “pancreatic rest” concept, which is perhaps the oldest postulate in the management of acute pancreatitis. As discussed in the preceding sections of this paper, a cornerstone of this concept is that avoidance or minimisation of the pancreatic enzyme secretory response might prevent exacerbation of the acute inflammatory process in the pancreas [40, 45, 105, 106]. At least in theory, this may be achieved by administration of a feeding formulation that does not require pancreatic enzymes for absorption (e.g., amino acids or oligopeptides). This was the reason why use of polymeric and fibre-enriched formulations was avoided for decades. However, only two prospective studies showed that a polymeric formulation increases pancreatic enzyme secretion into the duodenum in comparison with an elemental formulation, and both of these studies were conducted in healthy volunteers [67, 107]. Conversely, a randomised controlled trial of patients undergoing resection of the pancreas showed that a polymeric formulation did not increase pancreatic secretion compared with an elemental formulation [108]. The latter finding supports the results of the systematic review by Petrov and colleagues that demonstrated no increase in adverse effects with the use of polymeric and fibre-enriched formulations. Although one might argue that such an inference is premature and it is necessary to wait for a definitive randomised controlled trial, a power calculation shows that this is no simple undertaking. An adequately powered randomised controlled trial would need to enrol 1959 patients per study arm in order to demonstrate a 1.5% absolute reduction in the risk of death between the groups with 80% power and two-sided *α* = 0.05. Such a sample size appears to be unrealistically large, even for a multicentre trial.

It is also worth noting that elemental and polymeric feeding formulations were rigorously compared over the last two decades in a number of randomised controlled trials in patients with active Crohn's disease, which is perhaps the only disease, apart from AP, in which enteral feeding is used as an established key element of treatment [97, 109, 110]. By 1995, four randomised controlled trials were published, and subsequent meta-analyses failed to demonstrate a difference in efficacy between elemental and polymeric formulations [111]. By 2007, a total of 10 randomised controlled trials were published, but a Cochrane systematic review still found no difference in the induction of remission of active Crohn's disease when different formulations were compared [112]. Hence, it is argued that the use of polymeric feeding formulations is safe in patients with acute pancreatitis, and the research community may now focus on other issues in acute pancreatitis nutrition.

## 5. Timing of Enteral Nutrition

It is believed that gut dysfunction contributes to the inflammatory response and organ failure in severe acute pancreatitis. Theoretically, enteral feeding can prevent mucosal barrier dysfunction, small bowel bacterial overgrowth, and bacterial translocation and, therefore, should be instituted as early as possible in the course of disease [14, 15, 113, 114]. However, while some authors showed the clinical benefits of early enteral nutrition, others demonstrated the favourable effects of delayed enteral feeding [19, 29]. Unfortunately, such a strategical question as the timing of enteral nutrition in patients with acute pancreatitis has never been studied in randomised controlled trials. At the same time, some randomised controlled trials in critically ill patients suggest that enteral feeding start time has to be within hours of onset of disease.

In particular, burn patients were studied in the randomised controlled trial by Chiarelli and colleagues [115]. Patients were randomized to receive early enteral feeding within 4.4 ± 0.5 h postburn or delayed feeding administered a mean of 57.7 ± 2.6 h after injury. It was shown that patients with very early start of nutrition had fewer infections as well as a significantly shortened length of hospital stay. Furthermore, Graham and colleagues demonstrated with a randomised controlled trial the benefits of early (<36 h) enteral feeding compared with delayed (3–5 d) in 32 patients after head injury [116]. Infectious complications and length of hospital stay in the intensive care unit were reduced significantly with early feeding into the jejunum. In a randomised controlled trial conducted by Peng and colleagues, 22 patients with severe burns were randomized to either early enteral feeding (within 24 h) or delayed enteral feeding (after 48 h). The urinary lactulose levels and the urinary lactulose-mannitol ratios in the early group were significantly lower than in the delayed group as well as the levels of serum endotoxin and TNF-*α*. It was suggested that early enteral feeding may decrease intestinal permeability, preserve the intestinal mucosal barrier, and have a beneficial effect on the reduction of enterogenic infection. A recent randomised controlled trial from Slovenia in 52 patients with multiple injuries demonstrated that enteral nutrition administered on admission, as compared to enteral feeding started after 24 h of admission, was associated with a lower incidence of upper intestinal intolerance and nosocomial pneumonia. A meta-analysis by Marik and Zaloga demonstrated the benefits of early enteral nutrition (started from 2 to 24 h after operation) versus delayed feeding in terms of reducing episodes of infection and length of hospital stay in patients after abdominal surgery [117].

However, the usefulness of early onset of enteral feeding has not been shown in some other studies. In 2004, Peck and colleagues reported the results of their randomised controlled trial on 27 patients with burn injury. The study demonstrated that early enteral nutrition (<24 h of injury) had no beneficial effect on postburn hypermetabolism when compared with late (>7 d) enteral feeding and also did not result in a reduction of mortality, infectious complications, or hospital stay. In another trial, Dvorak and colleagues randomised 17 patients with acute spinal cord injury to early (initiated before 72 h after injury) or late (started more than 120 h after injury) enteral feeding. The randomised controlled trial failed to detect any differences in the incidence of infection, nutritional status, feeding complications, or length of stay between studied groups. Unfortunately, both randomised controlled trials were underpowered and thereby presented results should be interpreted with caution. Considering the results from randomised controlled trials on this subject in patients with surgical conditions, it seems that there is a sufficient body of evidence to state the need for high quality randomised controlled trial on the efficacy of early versus delayed enteral nutritional strategy in patients with severe acute pancreatitis.

## 6. Regimen of Enteral Nutrition

The impaired gastrointestinal motility is an important factor in the pathogenesis of complications of acute pancreatitis [51, 118–120]. It may lead to proximal stagnation, gastric and small intestinal bacterial overgrowth with subsequent bacterial translocation, and infection of pancreatic necrosis. Thereby, a recent study questioned the rationale of fasting patients with acute pancreatitis prior to oral refeeding and advocated the early enteral feeding to prevent or attenuate ileus. The logic behind early enteral nutrition is that feeding may stimulate motor migrating complex which is responsible for coordinated propulsive activity of the gastrointestinal tract. At the same time, gut hormones may have an important role in regulating gastrointestinal motility.

In particular, high cholecystokinin level is known to cause delay in gastric empting, and to regulate nutrient-induced jejunogastric feedback mechanism and, thereby, can have influence on the tolerance of enteral feeding [100, 103, 121]. Traditionally, continuous, as opposite to intermittent, EN has been recommended to increase the tolerance of nutrition. Nutritional diet is usually started at low rates (15 mL/h), with gradual advancement to ensure tolerance. However, this tactic has never been tested in randomised controlled trials. By contrast, it was reported in a randomised controlled trial on continuous versus cyclic jejunal nutrition in patients undergoing pylorus-preserving pancreatoduodenectomy that patients on cyclic (discontinuing the nutrition during the night) enteral nutrition had significantly lower levels of cholecystokinin during interruption of feeding. Clinically this finding was associated with shorter length of hospital stay (*P* < 0.05) and earlier resumption of oral diet (*P* < 0.05) in the intermittent group. In accordance with these data, a recent randomised controlled trial on continuous versus intermittent gastric feeding in critically ill trauma patients showed that the intermittent regimen patients (100 mL of enteral feed during a 30- to 60-minute period of time every 8 h) reached the nutritional goal faster (*P* = 0.01). However, there was no difference between groups in terms of complications and mortality.

The data mentioned above suggest that a randomised controlled trial on continuous versus intermittent enteral feeding may be of practical importance in patients with acute pancreatitis.

## 7. Further Directions

Findings from the randomised controlled trials discussed above have highlighted a number of major areas that require further investigations.

### 7.1. Further Randomised Trials of Nasogastric Tube Feeding in Patients with Acute Pancreatitis

**Figure 1:**
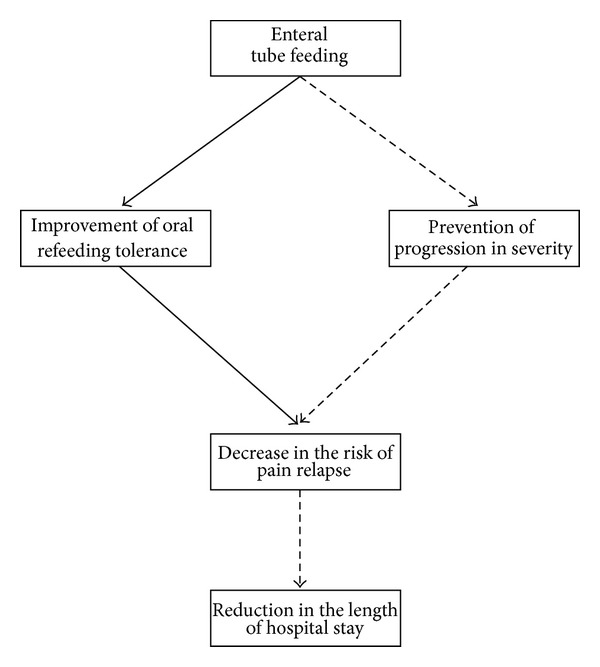
Hypotheses that need to be evaluated (dashed line) in further randomised controlled trials of nasogastric tube feeding in patients with acute pancreatitis.

Nasogastric tube feeding appears to be safe and well tolerated in the vast majority of patients with acute pancreatitis. However, the evidence base is limited, with only four rather small randomised controlled trials of nasogastric tube feeding. Larger-scale randomised controlled trials looking at the effect of nasogastric tube feeding in patients with acute pancreatitis are desirable. In particular, a quality randomised controlled trial is needed to investigate whether nasogastric tube feeding can prevent the progression of severity in patients with acute pancreatitis [122, 123]. The potential benefits which enteral nutrition may offer are dual, that is, improvement in tolerance of oral refeeding and prevention of progression in severity of acute pancreatitis (Figure 1). A further randomised controlled trial should also determine whether a decrease in the risk of pain relapse results in a statistically significant reduction in length of hospitalisation and, eventually, overall cost of treatment.

A particular emphasis in future studies must be in relation to study populations. Given that the use of predictive criteria of severity was advocated by the Atlanta classification, the majority of randomised controlled trials conducted to date have enrolled patients on the basis of various criteria of predicted severity with different thresholds (Ranson score > 3, Acute Physiology And Chronic Health Evaluation II score > 8, Acute Physiology And Chronic Health Evaluation II score > 7, Acute Physiology And Chronic Health Evaluation II score > 6, C reactive protein > 150, C reactive protein > 120). Unfortunately, the field is hampered by suboptimal definitions of that for which prediction is sought. A recent systematic review showed that there was remarkable heterogeneity between the studies in this regard [124]. The endpoints for the prediction of severity included multiple factor prognostic scores (Acute Physiology And Chronic Health Evaluation II ≥ 8 and/or Ranson ≥ 3), death, local, and/or systemic complications (as defined by the Atlanta symposium), Japanese criteria of severity, organ failure, pancreatic necrosis, infected pancreatic necrosis, length of hospitalisation, intensive care unit admission, and need for surgery. This is one of the main reasons why modern prognostic scores can, on average, correctly predict severity in only 60%–80% of patients. Moreover, a randomised controlled trial from a well-known group with an interest in acute pancreatitis employed an Acute Physiology And Chronic Health Evaluation II score ≥ 8 to enrol patients with a predicted severe course of acute pancreatitis and found that actual severe acute pancreatitis (as defined by the Atlanta symposium) occurred in only 46% [41]. That is inferior to tossing a coin and definitely more labour intensive and time consuming! The important implication for nutritional management of patients with acute pancreatitis is that the exact (actual) population of patients with acute pancreatitis who benefit from enteral nutrition is still largely unknown [125].

That is why future studies in the field should employ the new classification of severity, which is based on actual determinants of severity, to enrol patients in the trial and assess the effect of treatment [126–128]. This will ensure that nutritional management is tailored to patients with each category of actual severity. In particular, randomised controlled trials are needed to investigate the optimal nutritional management in the most challenging patients, that is, those with severe and critical acute pancreatitis. Given that these patients are not prevalent in routine practice, a multicentre (international) collaboration will be required.

### 7.2. Pilot Randomised Trial of Early Nasogastric Tube Feeding versus Oral Feeding Ad Libitum in Patients with Mild-to-Moderate Acute Pancreatitis

**Figure 2:**
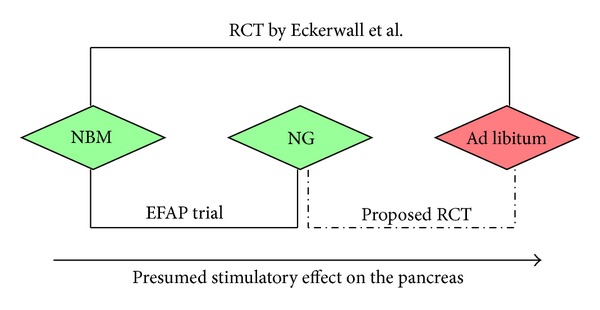
Place of proposed pilot RCT of early nasogastric tube feeding versus oral feeding ad libitum in the literature. NBM: nil-by-mouth; NG: nasogastric tube feeding; RCT: randomised controlled trial.

The benefits of early (within 24 hours of hospital admission) nasogastric tube feeding in comparison with a nil-by-mouth regimen were demonstrated in the EFAP (Enteral Feeding in Acute Pancreatitis) trial [129]. This randomised trial showed that early administration of nasogastric tube feeding is safe and does not exacerbate the course of acute pancreatitis. Furthermore, the use of nasogastric tube feeding significantly reduces the intensity and duration of initial pain and prevents pain relapse after oral refeeding. At the same time, a recent randomised controlled trial from Sweden compared early (within 24 hours of hospital admission) oral feeding ad libitum with nil-by-mouth and showed that oral feeding does not exacerbate the course of acute pancreatitis and even reduces the total length of hospital stay, but no significant effect on the intensity and duration of initial pain was observed [96]. A pilot randomised controlled trial is now warranted to compare directly the interventions used in the two trials, that is, nasogastric tube feeding and oral feeding ad libitum (Figure 2). A randomised controlled trial of this design would be of both practical and theoretical importance. From a practical perspective, it would help to determine the optimal early feeding regimen in patients with acute pancreatitis. From a theoretical perspective, it would provide a definitive answer as to whether the “pancreas rest” concept can be buried or not.

### 7.3. Definitive Randomised Controlled Trial of Fibre-Enriched Enteral Nutrition in Acute Pancreatitis

Enteral nutrition is associated with a relatively high rate of adverse effects in patients with acute pancreatitis, in particular diarrhoea. A meta-analysis of 13 randomised controlled trials comparing fibre-enriched and fibre-free enteral feeding formulations showed a significant reduction of diarrhoea in patients receiving the former [45]. This makes fibre-enriched formulations a promising and clinically relevant approach to minimise the risk of diarrhoea in patients with acute pancreatitis. To date, the effect of fibre has been evaluated in only one randomised controlled trial in patients with predicted severe acute pancreatitis [85]. That study demonstrated no cases of diarrhoea in 15 patients receiving a fibre-enriched formulation, compared with two of 15 patients receiving a fibre-free formulation. Taking into account the limited sample size of the study, an adequately powered randomised controlled trial of a fibre-enriched versus fibre-free formulation in patients with acute pancreatitis appears to be warranted.

The call for such a trial was also supported by the eagerly awaited and very disappointing results of the PROPATRIA trial [95]. A common interpretation of this study is that the use of probiotics in patients with predicted severe AP led to a significantly increased risk of intestinal ischaemia, multiple organ failure, and mortality. However, this is not entirely correct because, in fact, the intervention group in the PROPATRIA received a fibre-enriched formulation supplemented with six strains of probiotics, whereas the control group received a fibre-enriched formulation alone. Therefore, one can only conclude from this study that the given combination of probiotics and fibres was harmful, whereas the fact that mortality was only 6% in the control group and that no cases of intestinal necrosis were observed, shows that a fibre-enriched formulation is safe in patients with acute pancreatitis.

### 7.4. Further Studies on Immunonutrition in Acute Pancreatitis

To date, only three randomised controlled trials on the use of immunonutrition are available in the literature, and they did not demonstrate any clinical beneficial effect of enteral nutrition enriched with glutamine, arginine, and/or omega-3 fatty acids when compared with standard enteral nutrition in patients with acute pancreatitis [92, 130]. At first sight, given that the sample sizes of all the randomised controlled trials were too small, it seems that no definitive conclusions can be drawn from them. Nevertheless, an authoritative meta-analysis of several hundred critically ill and elective surgery patients, which was greater in terms of study population, found a statistically significant benefit of immunonutrition (reduced risk of infectious complications) only in the subgroup of patients who received a high-arginine-content formula. However, excessive arginine supplementation could have a potentially damaging effect on the pancreas, probably due to the excessive production of nitric oxide. It is also known that administration of omega-3 fatty acids decreases antioxidant capacity [131]. Nevertheless, the relevance of these experimental observations is difficult to evaluate in the clinical setting because immunonutrition is usually administered in a compound and it is hard to ascribe a beneficial or harmful effect of an immunonutritional formulation to any single immune-enhancing agent. Thus, further clinical and animal studies should focus on individual immune-enhancing agents, of which glutamine seems to have the greatest beneficial potential in the setting of acute pancreatitis.

### 7.5. Pilot Study of Antioxidant-Enriched Enteral Nutrition in Acute Pancreatitis

Although oxidative stress has been implicated as an important factor in the pathogenesis of acute pancreatitis for nearly three decades, the therapeutic effect of antioxidants in patients with acute pancreatitis has been investigated in only a few inconclusive randomised controlled trials. The only high-quality trial enrolled 43 patients with predicted severe acute pancreatitis (Acute Physiology And Chronic Health Evaluation II score ≥ 8 within 48 hours of admission) and showed no benefit of intravenous antioxidant therapy (n-acetylcysteine, selenium, and vitamin C) administered for 7 days [132]. Moreover, the study was terminated at the time of interim analysis because some data suggested that the intervention might even be harmful. Following the publication of this study in 2007, some authors rushed to conclude that “the book on the antioxidant story in the treatment of acute pancreatitis has closed” [133]. However, while the trial clearly showed that the given combination of antioxidants is not effective in patients with acute pancreatitis when administered intravenously, it is quite possible that antioxidants administered via another route will be beneficial. In particular, there is a growing body of clinical evidence from other disease settings that supplementation of enteral nutrition with antioxidants might be beneficial. Given that the benefits of standard enteral nutrition in patients with acute pancreatitis are well proven, the use of antioxidant-enriched enteral nutrition may be a sensible direction of clinical research in acute pancreatitis.

## 8. Conclusion

The frontiers in nutritional management of patients with acute pancreatitis continue to advance. The findings presented in this review highlight the importance of enteral nutrition in curtailing of acute inflammation of the pancreas. There is ample evidence in the literature that the use of nasojejunal tube feeding improves outcomes in patients with predicted severe course of acute pancreatitis. Several studies have demonstrated the safety and efficacy of nasogastric tube feeding in these patients. Furthermore, for the first time, it has been shown in a randomised trial that early nasogastric tube feeding may have benefits for patients with actual mild-to-moderate acute pancreatitis. Lastly, optimal enteral feeding formulations have been determined based on the best available data. Further expansion of frontiers in nutritional management of acute pancreatitis represents a formidable opportunity for improving patient care. As long ago as 1928, Bertrand Russell wrote “*the extent to which beliefs are based upon evidence is very much less than believers suppose*.” This still pretty much holds true today in the management of the most frequent disease of the pancreas.

## Figures and Tables

**Table 1:** Randomised controlled trials of total enteral versus total parenteral nutrition in patients with predicted severe acute pancreatitis.

Reference	Year	Setting	Patients (*n*)		Allocation concealment	Reduction of infectious complications and mortality
			Enteral nutrition	Parenteral nutrition		
Kalfarentzos et al. [70]	1997	Greece	18	20	Openlabel	Significantly lower rate of pancreatic infection in the total enteral nutrition group
Gupta et al. [43]	2003	UK	8	9	Openlabel	Non-significantly lower rate of pancreatic infection in the total enteral nutrition group
Louie et al. [71]	2005	Canada	10	18	Openlabel	Non-significantly lower rate of pancreatic infection in the total enteral nutrition group
Eckerwall et al. [41]	2006	Sweden	23	25	Openlabel	No significant difference in outcomes
Petrov et al. [72]	2006	Russia	35	34	Openlabel	Significantly lower rate of pancreatic infection and mortality in the total enteral nutrition group
Casas et al. [73]	2007	Spain	11	11	Openlabel	Non-significantly lower rate of pancreatic infection in the total enteral nutrition group
Doley et al. [74]	2008	India	25	25	Openlabel	No significant difference in the outcomes
Wu et al. [75]	2010	China	53	54	Openlabel	Significantly lower rate of pancreatic infection and mortality in the total enteral nutrition group

**Table 2:** Characteristics of studies of nasogastric tube feeding.

Reference	Setting	Design	Control group	APACHE II Score	Feeding start	Feeding formulation	Duration of nutrition	Quality of studies^§^
Eatock et al. 2000 [76]	UK	Cohort study	N/A	10 (4–28)^#^	<48 hours of admission	Semielemental	Not stated	N/A
Eatock et al. 2005 [58]	UK	RCT	Nasojejunal	10 (7–18)^#^	72 (24–72) hours after onset	Semielemental	5 days	14
Kumar et al. 2006 [59]	India	RCT	Nasojejunal	10.5 ± 3.8^‡^	48–72 hours of admission	Semielemental	7 days	13
Eckerwall et al. 2006 [41]	Sweden	RCT	Parenteral	10 (8–13)^#^	<24 hours of admission	Polymeric	6 (5–9)^#^ days	14
Singh et al. 2012 [64]	India	RCT	Nasojejunal	8.5 (2–19)^#^	10 (4–23)^#^ days after onset	Semielemental	7 days	13

^§^Range of quality score is 0 to 16; ^#^values are median (range); ^‡^values are mean ± standard deviation. Abbreviations: APACHE: Acute physiology and chronic health evaluation; RCT: randomised controlled trial; N/A: not available.

**Table 3:** Characteristics of patients receiving nasogastric tube feeding.

Reference	Age	Male : female	Aetiology		
			Biliary	Alcohol	Other
Eatock et al. 2000 [76]	47 (27–96)^#^	12 : 14	18	5	3
Eatock et al. 2005 [58]	63 (47–74)^#^	14 : 13	16	6	5
Kumar et al. 2006 [59]	43.3 ± 12.8^‡^	14 : 2	8	4	4
Eckerwall et al. 2006 [41]	71 (58–80)^†#^	10 : 14^†^	14^†^	3^†^	7^†^
Singh et al. 2012 [64]	39.1 ± 16.7^‡^	28 : 11	12	12	15

^†^Before exclusion of protocol violator (one patient); ^#^values are median (range); ^‡^values are mean ± standard deviation.

**Table 4:** Safety and tolerance of nasogastric tube feeding.

Reference	Total patients	Troublesome diarrhoea *n*, (%)	Tube removal *n*, (%)	Gastric retention *n*, (%)	Exacerbation of pain following feeding, *n*, (%)	Achievement of nutritional goal	Full tolerance of feeding *n*, (%)^†^
Eatock et al. 2000 [76]	26	3 (11.5)	1 (3.8)	3 (11.5)	0 (0)	Not stated	19 (73.1)
Eatock et al. 2005 [58]	27	3 (11.1)	1 (3.7)	0 (0)	2 (7.4)	21 patients (78%) after 60 hours	23 (85.1)
Kumar et al. 2006 [59]	16	4 (25)	1 (6.3)	0 (0)	1 (6.3)	16 patients (100%) by day 7*	11 (68.8)
Eckerwall et al. 2006 [41]	23	0 (0)	0 (0)	3 (13)	Not stated	15 patients (66%) by day 7	20 (86.9)
Singh et al. 2012 [64]	39	4 (10.4)	0 (0)	1 (2.5)	3 (7.7)	Not stated	34 (85.6)

Total	131	14 (10.7)	3 (2.3)	7 (5.3)	6 (4.5)	N/A	107 (82.0)

^†^Did not require temporary reduction, stoppage, or withdrawal of feeding; *six patients were supplemented by parenteral nutrition during the commencement of feeding. N/A: not available.

**Table 5:** Outcomes of patients who received nasogastric feeding.

Reference	Total patients (*n*)	Patients on ventilatory support *n*, (%)	Patients with MOF *n*, (%)	Infected pancreatic necrosis *n*, (%)	Surgery *n*, (%)	Mortality *n*, (%)	LOS, days
Eatock et al. 2000 [76]	26	11 (42.3)	6 (23.1)	5 (19.2)	10 (38.5)	4 (15.4)	17.5 (3–82)
Eatock et al. 2005 [58]	27	7 (25.9)	Not stated	Not stated	Not stated	5 (18.5)	16 (10–22)
Kumar et al. 2006 [59]	16	15 (93.8)	3 (18.8)	5 (31.3)	1 (6.3)	5 (31.3)	24 ± 14.3
Eckerwall et al. 2006 [41]	23	2 (8.7)	1 (4.3)	1 (4.3)	1 (4.3)	1 (4.3)	9 (7–14)
Singh et al. 2012 [64]	39	26 (66.7)	11 (28.2)	4 (10.2)	4 (10.2)	4 (10.2)	17 (1–73)

Total	131	61 (46.5)	21 (16.0)	15 (11.5)	16 (12.2)	19 (14.5)	N/A

MOF: multiple organ failure; LOS: length of hospital stay; NA: not available.

**Table 6:** Characteristics of randomised controlled trials of various enteral nutrition formulations.

Reference	Year	Intervention group	Control group	Number of patients			
				Intervention group	Control group	Severe	Mild
McClave et al. [77]	1997	Semielemental EN	PN	15	15	6	24
Kalfarentzos et al. [70]	1997	Semielemental EN	PN	18	20	38	0
Windsor et al. [42]	1998	Polymeric EN	PN	16	18	13	21
Powell et al. [40]	2000	Polymeric EN	NN	13	14	27	0
Hallay et al. [78]	2001	EN with fibre + glutamine + arginine	EN with fibre	11	8	19	0
Olah et al. [79]	2002	Elemental EN	PN	41	48	17	72
Abou-Assi et al. [80]	2002	Elemental EN	PN	26	27	26	27
Olah et al. [81]	2002	EN with fibre + probiotics	EN with fibre	22	23	32	13
Gupta et al. [43]	2003	Polymeric EN	PN	8	9	17	0
Louie et al. [71]	2005	Semielemental EN	PN	10	18	28	0
Lasztity et al. [82]	2005	Polymeric EN + n-3 PUFAs	Polymeric EN	14	14	6	22
Pearce et al. [83]	2006	EN with fibre + glutamine + arginine + omega-3 fatty acids	EN with fibre	15	16	31	0
Tiengou et al. [84]	2006	Semielemental EN	Polymeric EN	15	15	19	11
Eckerwall et al. [41]	2006	Polymeric EN	PN	23	25	48	0
Petrov et al. [72]	2006	Semielemental EN	PN	35	34	69	0
Casas et al. [73]	2007	Semielemental EN	PN	11	11	22	0
Olah et al. [80]	2007	EN with fibre + probiotics	EN with fibre	33	29	62	0
Karakan et al. [85]	2007	EN with fibre	Polymeric EN	15	15	30	0
besselink et al. [86]	2008	EN with fibre + probiotics	EN with fibre	152	144	296	0
Qin et al. [87]	2008	Semielemental EN + probiotics	PN	36	38	19	55

EN: enteral nutrition; PN: parenteral nutrition; PUFA: polyunsaturated fatty acids.

**Table 7:** Pooled estimates and sensitivity analysis.

Comparison	Severity of acute pancreatitis	Feeding intolerance		Total infectious complications		Mortality	
		RR (95% CI)	*P*	RR (95% CI)	*P*	RR (95% CI)	*P*
(Semi)-elemental versus polymeric	Mild or severe	0.62 (0.10–3.97)*	0.61	0.48 (0.06–3.76)*	0.48	0.63 (0.04–9.86)*	0.74
	Severe only	2.26 (0.32–15.27)*	0.41	0.23 (0.03–1.86)*	0.25	0.89 (0.28–4.90)*	0.12

Fibre-enriched + probiotics versus fibre-enriched	Mild or severe	0.69 (0.43–1.09)^#^	0.11	0.71 (0.40–1.27)^#^	0.25	0.85 (0.18–4.14)^#^	0.85
	Severe only	0.69 (0.43–1.09)^#^	0.11	0.79 (0.40–1.56)^#^	0.50	0.96 (0.12–7.83)^#^	0.97

Fibre-enriched + immunonutrition versus fibre-enriched	Mild or severe	1.60 (0.31–8.29)^#^	0.58	0.93 (0.36–2.40)^#^	0.88	0.60 (0.10–3.55)^#^	0.58
	Severe only	1.60 (0.31–8.29)^ #^	0.58	0.93 (0.36–2.40)	0.88	0.60 (0.10–3.55)^#^	0.58

*Indirectly estimated RR and its 95% CI; ^#^directly estimated RR and its 95% CI. CI: confidence interval; RR: relative risk.
